# Xeroderma Pigmentosum

**DOI:** 10.1093/omcr/omab107

**Published:** 2021-11-25

**Authors:** Gautam Srivastava, Govind Srivastava

**Affiliations:** 1 Faculty of Life Sciences and Education, University of South Wales, UK; 2 Department of Dermatology and Venereology, Skin Institute and School of Dermatology, New Delhi, India

A 25-year-old female came with increasingly pigmented spots on her face, arms, hands and feet from 2 years of age. She had taken multiple medications and was advised to avoid sunlight and wear full protective clothing. Despite these measures, her symptoms kept getting worse. She also frequently suffered from dry, red eyes and photophobia (Figure 1a). On examination, she had gross signs of actinic damage on exposed parts of the body along with conjunctival pigmentation and corneal opacity (Figure 1b). The lips had numerous lentigines and ephelides with no involvement of tongue and oral mucosa. There was suspected consanguinity in her family, although no such manifestations were reported before. A diagnosis of xeroderma pigmentosum (XP) was made. She was advised strict sun protection/avoidance, vitamin D supplementation and was referred to an ophthalmologist for ocular complaints. Further, she was advised biannual visits to a dermatologist and an ophthalmologist, and annual visits to a neurologist to prevent any neuro-degenerative symptoms in the future.

**Figure 1:**
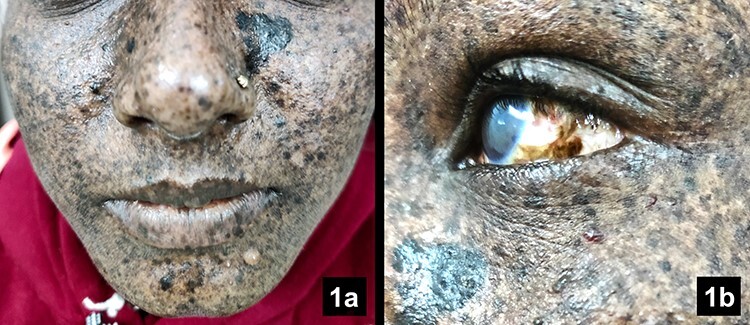
a) Face predominantly showing widespread ephelides, lentigines, keratoacanthoma and actinic keratoses overlying a grossly photodamaged facial skin. There is suspected malignant melanoma near the ala of the nose on the left side. b) Left eye showing conjunctival pigmentation, corneal opacity and early cataract.

XP is a rare autosomal recessive genodermatosis characterized by defective DNA repair resulting in extreme sensitivity to UV radiations. These patients are very prone for the development of skin malignancies and blindness [1]. First described by Moritz Kaposi in 1874, this entity was subsequently expanded to include eight subtypes—XP type A to G and XP-variant (XPV) [2]. There is no cure for XP but constant UV radiation protection of skin and eye, vitamin D supplementation to compensate for sun avoidance, systemic retinoid therapy in select cases, and early resection of pre-malignant and malignant lesions can improve prognosis and prolong life. Genetic counselling in young adults is important to highlight the potential risks to their offspring and explore the possible reproductive options. Recently, Lehmann and Fassihi [3] postulated that molecular analysis can improve the management, prognosis and therapy for individuals with XP.

## CONFLICT OF INTEREST STATEMENT

None declared.

## ETHICAL APPROVAL

Not applicable.

## CONSENT

The authors confirm that written consent for the publication of this case including images and associated text has been obtained from the patient.

## GUARANTOR

Dr Govind Srivastava is the guarantor for this publication.
